# Cost-effectiveness evaluation of carer-assisted home-based therapy versus usual therapy for long term stroke survivors in Malaysia

**DOI:** 10.1186/1471-2458-14-S1-O12

**Published:** 2014-01-29

**Authors:** Nor Azlin Mohd Nordin

**Affiliations:** 1 United Nations University International Institute for Global Health (UNU-IIGH), 56000 Cheras, Kuala Lumpur, Malaysia

## Background

There has been scarcity of research to assess the cost-effectiveness of rehabilitation interventions for post-stroke cases despite the increasing importance of this service to minimise post-stroke functional problems. The aim of this study was to evaluate the cost-effectiveness of a carer-assisted home-based therapy against a usual therapy for long-term stroke survivors residing in the community.

## Materials and methods

A randomised controlled trial was conducted in 91 stroke survivors who received either a carer-assisted home-based therapy or a therapist-directed hospital-based therapy (usual therapy) for 12 weeks. The therapy outcomes were measured in the aspects of physical functions and quality-adjusted life year (QALY) gained. The costs of the therapies were analysed from the health care provider’s and the patient’s perspectives, using a top-down costing approach. Cost-effectiveness of the therapies was estimated in terms of cost per unit of function gained and cost per QALY gained.

## Results

The functional outcomes and QALYs gained through the carer-assisted home-based therapy was comparable to the outcomes of the usual intervention, with gains up to 25% (p <0.05) following therapy. The carer-assisted therapy incurred 42% less costs when compared to the usual therapy, with mean cost saved for every stroke survivor amounted to about RM560 for the 12 weeks intervention. The therapy was found to be highly cost-effective, with incremental cost-effectiveness ratio (ICER) of RM5,619 per QALY gained, equivalent to 18% of the country’s per-capita gross domestic product.

## Conclusions

The findings support the use of a carer-assisted home-based therapy as a cost-saving strategy in the rehabilitation of people with long-term stroke. The therapy should be included in a long-term care plan for stroke survivors living in the community.

